# Associations Between Social Media, Bedtime Technology Use Rules, and Daytime Sleepiness Among Adolescents: Cross-sectional Findings From a Nationally Representative Sample

**DOI:** 10.2196/26273

**Published:** 2021-09-15

**Authors:** Jessica Leigh Hamilton, Woanjun Lee

**Affiliations:** 1 Department of Psychology Rutgers University Piscataway, NJ United States

**Keywords:** adolescents, social media, daytime sleepiness, parenting, bedtime, mental health, mobile phone

## Abstract

**Background:**

Social media use is associated with poor sleep among adolescents, including daytime sleepiness, which affects adolescents’ mental health. Few studies have examined the associations among specific aspects of social media, such as frequency of checking and posting, perceived importance of social media for social belonging, and daytime sleepiness. Identifying whether certain adolescents are more at risk or protected from the effects of social media on sleepiness may inform future interventions for social media, sleep, and mental health.

**Objective:**

This study aims to examine the association between social media use frequency and importance, daytime sleepiness, and whether the perceived importance of social media for social interactions and parental rules around bedtime technology moderated these relationships.

**Methods:**

This cross-sectional survey study was conducted with a sample of 4153 adolescents from across the United States. Qualtrics was used to collect data via panel recruitment from a national sample representing the US demographics of teens aged 12 to 17 years. Participants completed measures of daytime sleepiness, frequency of social media checking and posting, and the importance of social media for social interactions. Parents reported whether they had a household rule around bedtime media and screen use. Hierarchical regressions and moderation analyses were conducted, covarying for age, gender, and age at first smartphone use.

**Results:**

Participants had a mean age of 14.64 (SD 1.66) years in grades 6 to 12, 46.45% (1929/4153) identified as female, and 67.93% (2821/4153) identified as White. The results indicated that adolescents who posted (B=0.70, SE 0.04; *P*<.001) or checked (B=0.76, SE 0.04; *P*<.001) social media more frequently or who perceived social media to be more important for social belonging (B=0.36, SE 0.02; *P*<.001) had higher levels of daytime sleepiness. Moderation analyses indicated that the relationship between social media use frequency and daytime sleepiness was exacerbated by higher levels of perceived social media importance (B=0.04, SE 0.01; *P*<.001). Adolescents without household rules around bedtime technology use were more likely to be affected by social media checking (B=−0.34, SE 0.09; *P*<.001) and importance (B=−0.16, SE 0.04; *P*<.001) on daytime sleepiness.

**Conclusions:**

The findings suggest that social media use frequency and perceived importance of social interactions are associated with daytime sleepiness among adolescents. It is important to consider youth’s perceptions of social media when assessing the potential effects of social media use frequency on youth well-being. Furthermore, youth who did not have parental rules around bedtime technology use were most likely to be affected by social media use and perceived importance. The findings may extend to other mental health outcomes and may guide future prevention and intervention programs designed to improve social media use, sleep, and mental health.

## Introduction

### Background

Sleep health is critical for the promotion of adolescent mental health. One major consequence of poor sleep and insufficient sleep is daytime sleepiness [1]. Daytime sleepiness is common among adolescents [2-5] and has significant consequences for daytime functioning and mood. Adolescents who report excessive daytime sleepiness (ie, tendency to doze off or fall asleep during the day) are at risk for poorer academic performance, accidents and injuries, substance use, and mental health problems [6]. In particular, daytime sleepiness is linked with depression and suicidal thoughts and behaviors [7,8]. A recent longitudinal study found that excessive daytime sleepiness predicted future suicidal thoughts and attempts among adolescents [9]. Given the increase in the rates of adolescent depression and suicide [10], it is critical to move toward prevention. Thus, identifying modifiable behavioral factors that contribute to poor sleep health, and specifically, daytime sleepiness, may have upstream effects on promoting mental well-being and preventing adolescent depression and suicide.

Adolescents undergo normative developmental shifts in sleep-wake processes, including delayed circadian rhythms and decreasing homeostatic sleep drive (ie, *the need to sleep*), which shifts the natural propensity toward sleepiness to later in the evening [1,11,12]. Psychosocial factors, such as academic demands and heightened importance of engaging and socializing with peers [13], also shift sleep times later [12]. Portable electronic devices have further complicated this issue by providing adolescents with continuous, round-the-clock access to engaging and stimulating activities. Indeed, electronic device use among adolescents is associated in both cross-sectional and longitudinal studies with a range of sleep outcomes, including later, shorter, and poorer sleep [14-17], as well as daytime sleepiness [18-22]. Portable devices now offer teens access to communicate directly and indirectly with peers at any time of the day or night via social media. In the context of adolescent development, it is not surprising that social media is uniquely engaging for adolescents. Adolescence is a developmental stage in which there is an increasing importance of peer relationships and focus on social standing within ones’ peer groups [23]. Furthermore, neurobiological changes that occur in the adolescent brain heighten sensitivity to information and experiences with peers, including those that are socially rewarding and socially threatening [24,25]. Thus, adolescents can now engage with peers and access social information through social media’s unique and readily available platforms. Consequently, emerging research has sought to better understand the effects of social media on youth mental health [26].

With more than 90% of adolescents using social media, predominantly accessed through portable devices, adolescents are able to access social media at night, which is linked to later sleep onset, shorter sleep duration, and poorer sleep quality [27-30]. Several studies have also indicated a cross-sectional relationship between social media use and daytime sleepiness among adolescents [31,32]. However, few studies have examined whether specific aspects of social media use affect sleep outcomes, such as how adolescents use social media (eg, checking and posting behaviors) and individuals’ perceived importance of using social media for social connection. social media can provide adolescents with a range of social affordances, including a sense of belonging and a network of peers [33]. Although social media can have benefits for youth development and mental health [26,34], it may affect adolescent sleep behaviors and daytime sleepiness, especially for those who place heightened importance of using social media for social belonging may spend more time using social media. Thus, youth with both higher levels of social media use and greater perceived importance of social media for social connection may be doubly at risk for poor sleep outcomes, including daytime sleepiness. Identifying whether social media behaviors and perceived importance of social media affect daytime sleepiness and whether youth with both higher levels of social media use and perceived importance of social media are at greater risk may shed light on which aspects of social media are associated with sleepiness and guide future interventions around social media use and mental health.

To date, most studies have examined the relationship between social media and mental health in isolation without considering contextual factors that may influence the effects of social media use. Despite developmental shifts toward peers during adolescence, parents continue to play an important role in adolescents’ lives [35]. In particular, parental rules around bedtime affect youth sleep behaviors and sleep duration [36-38]. Specific behaviors or rules around digital media, particularly around bedtime, may buffer the potential negative effects of social media on daytime sleepiness among adolescents. Indeed, one study found that parents’ rules about technology use at bedtime were associated with an earlier bedtime for adolescents [39]. However, this study only reported the effect of technology rules on adolescents’ sleep without considering the extent to which youth use social media or their perceived importance of social media. It is critical to better understand whether parents’ technology rules, particularly around bedtime, affect the effect of social media on daytime sleepiness, which may provide valuable information for improving youth well-being and mental health.

### Objectives

The primary aim of this study is to examine the association between social media, including frequency of social media behaviors (posting and checking), perceived importance of social media for social interactions, and daytime sleepiness. It was hypothesized that adolescents with more frequent social media behaviors and greater importance of social media for social connection would have higher levels of daytime sleepiness. However, it is critical to identify *which youth* are most vulnerable to the effects of social media on sleepiness, which is a risk factor for poor mental health outcomes [1]. Thus, the second and third aims of this study are to evaluate whether (1) social media importance moderated the effect of social media frequency and daytime sleepiness and (2) whether parental bedtime technology rules moderated the effects of social media (frequency and importance) on daytime sleepiness. It was hypothesized that youth with greater perceived social media importance and more frequent social media behaviors would have greater daytime sleepiness. Furthermore, it was expected that the effect of social media on daytime sleepiness would be exacerbated among youth with no parental rules around bedtime media use.

## Methods

### Recruitment and Procedures

A cross-sectional web-based survey was conducted using the Qualtrics platform, which was used for both participant recruitment and survey administration. Potential participants were recruited by Qualtrics through web-based advertisements on social media and in-app games. Adolescents and their parents or legal guardians were recruited using the following criteria: adolescents aged 12 to 17 years and English speakers. Adolescents were recruited to be a representative sample of the race and ethnicity of the 2010 US census population for 12- to 17-year-olds. Qualtrics’ recruitment procedures took place between February and April 2019 and included a background check to confirm participants’ eligibility. In this study, only adolescents who reported being in grades 6 to 12 were included, as school environments may affect sleep opportunities and daytime sleepiness. Adolescents’ legally authorized representatives provided informed consent, and adolescents provided informed assent before being instructed to complete the web-based survey in a location that was private or where they felt comfortable. Adolescents were compensated if they completed at least 50% of the survey, and those who did not complete the survey were automatically replaced by Qualtrics recruitment. Participants were compensated by earning credit toward rewards (eg, gift cards and in-app purchases). This survey was approved by a local institutional review board.

### Survey Measures

#### Daytime Sleepiness

The eight-item Pediatric Daytime Sleepiness Scale was used to assess daytime sleepiness [40]. Participants answered items on a 5-point Likert scale, ranging from 0 (never) to 4 (always). Item examples include, *How often do you fall asleep or get drowsy during class periods?*, *How often do you need someone to awaken you in the morning?,* and *How often do you think that you need more sleep?* The internal consistency in this study was 0.85.

#### Social Media Behavior Frequency and Importance

Social media behavior frequency was assessed using two items: checking and posting behaviors. Items included *How often do you check social media?* and *How often do you post on social media?*, with response options ranging from *Never* to *Almost Constantly*. Items were examined separately, and scores were reverse coded so that higher values reflect more frequent social media behaviors. For these items, social media was not defined for participants. Examples of checking might include opening the social media to browse others’ updates or checking likes or comments on a post. Examples of posting might include posting photos or updates on social media. To understand the quality of social media use, adolescents completed the Adolescents’ Digital Technology Interactions and Importance Scale [41]. The Adolescents’ Digital Technology Interactions and Importance Scale is an 18-item measure that assesses the importance of activities on social media to bridge online and offline preferences and experience, go outside one’s identity in the offline environment, and social connection (eg, see what people are up to without asking them, direct message, and video chat). Given the unique importance of social connection during adolescence and the use of social media for social affordances [33], the importance of *technology for social connection* was the focus of this study (5 items). Adolescents responded to questions regarding the importance of their engagement in these activities on a 5-point Likert scale, ranging from *Not at all important* to *Extremely important*. Scores were summed for each category, with higher scores reflecting greater perceived importance. The internal consistency of the technology for the social subscale was 0.92.

#### Household Technology Rules

Parents reported household rules around media and technology by endorsing whether they have certain rules. For this study, the primary focus was whether a bedtime technology rule was endorsed (yes or no): “My house has rules about viewing screens around bedtime.” Parents also reported whether they had rules around *screen-free* zones and *screen-free times* (yes or no). To compare specificity to bedtime rules (vs total media rules), a total score was created to reflect the total number of rules involving media access, with scores ranging from 0 to 3. Age at first smartphone use also was assessed via both parent and teen report (ie, “How old were you or your child when you received your first smart phone [a phone that is able to connect to the internet]?”), which was used as a covariate in this study.

### Statistical Analyses

Descriptive analyses were first conducted using two-tailed independent *t* tests and correlations to evaluate demographic differences by age and gender. Regression analyses were conducted using R software (version 3.6; R Core Team). The analyses examined the direct relationships between the frequency of social media behaviors (checking and posting, separately) and daytime sleepiness and the importance of social media for social connection and daytime sleepiness. Given the similar patterns of findings for direct effects and the high correlation between social media checking and posting behaviors, an average score of social media frequency (posting and checking) was used for all interaction analyses to be more parsimonious. Moderation analyses were conducted to examine the interaction between social media frequency and the importance of social media. The interactions between social media (average frequency and importance, separately) and endorsement of bedtime technology rules were also tested. To determine the specificity of bedtime technology rules compared with having more rules around access to media, an interaction was included between social media and the total number of media access rules. Continuous predictors were centered before the analyses. Significant interactions for continuous variables were probed at one SD above or below the mean to reflect high and low levels of social media importance or at each level for household rules. Covariates included age, gender, and parent-reported age at first smartphone use. A total of eight regressions were conducted, and Bonferroni corrections were used to control for multiple comparisons (*P=.*006). The effect sizes (changes in *r^2^*) were calculated for all analyses.

## Results

### Participants

The final sample included 4153 adolescents, with a mean age of 14.64 (SD 1.66) years. Adolescents were evenly distributed across ages of 12 to 17 and grades 6 to 12, with the majority (2177/4153, 52.42%) in grades 8 to 10. Per the study design, the sample was evenly divided by gender and reflected the demographic characteristics of the United States for ages 12 to 17 years (Table 1). The legally authorized guardians of adolescent participants included 86.01% (3572/4153) biological parents and 13.99% (581/4153) were the adolescents’ foster or adoptive parents, grandparents, or other relatives or guardians. A total of 58.01% (2409/4153) of parents (inclusive of all guardians) were female, and 92.01% (3821/4153) obtained at least a high school education.

**Table 1:** Demographic information for the sample (N=4153)^a^.

Variables			Full sample statistics
**Gender, n (%)**			
	Female	1929 (46.45)	
	Male	2184 (52.59)	
	Nonbinary or transgender	35 (0.84)	
**Race, n (%)**			
	White	2821 (67.93)	
	Black or African American	608 (14.64)	
	Asian	210 (5.06)	
	American Indian, Alaska Native, Native Hawaiian, or Other Pacific Islander	130 (3.13)	
	Multiracial	205 (4.94)	
**Ethnicity, n (%)**			
	Hispanic or Latino	733 (17.65)	
Age (years), mean (SD)			14.64 (1.66)
Grade, mean (SD)			8.58 (1.78)
**Parent education, n (%)**			
	High school or less	249 (6)	
	High school or General Educational Development	782 (18.83)	
	Some college or associate’s	1321 (31.81)	
	Four-year college or bachelor’s	967 (23.28)	
	Professional degree	810 (19.5)	
**Sleepiness and social media behaviors, mean (SD)**			
	Daytime sleepiness	13.08 (6.62)	
	Social media checking frequency	5.00 (2.64)	
	Social media posting frequency	5.79 (2.38)	
	Technology for social importance	16.26 (5.57)	
**Technology access and rules**			
	Bedtime technology rules (yes), n (%)	1417 (34.12)	
	Total media access rules, mean (SD)	1.03 (1.06)	
	Smartphone age (parent), mean (SD)	12.36 (2.19)	
	Smartphone age (teen), mean (SD)	11.98 (2.01)	

^a^Participant responses that were not provided or stated prefer not to say are not included in the above table.

### Descriptive Results

Nearly all (3987/4153, 96%) adolescents and their parents reported that they own a smartphone. Parents and teens reported that their first smartphone use was at the age of approximately 12 years. Parents and teen reports correlated at 0.81. Of teens who owned smartphones, parents reported that 8.83% (352/3987) owned their first phone before the age of 10, 21.77% (868/3987) owned their first phone at ages 10 or 11, 41.03% (1636/3987) owned their first phone at ages 12 or 13, and 28.37% (1131/3987) owned their first phone at age ≥14 years. A total of 34.12% (1417/4153) of parents reported having rules around bedtime media or screen time, 41.15% (1709/4153) of parents reported having no media access rules, and 13.85% (575/4153) of parents reported having all three rules around media access. In addition, 23.24% (965/4153) of teens reported checking social media constantly, and approximately 12.62% (524/4153) of teens reported posting constantly. Teens’ posting and checking behavior was significantly correlated (r=0.80), as were reports of interest and importance of social media use (r*=*0.55-0.57). Teens with parental bedtime rules had lower social media frequency of posting and checking (t_4151_=8.94 and t_4151_=7.33, respectively; *P*<.001) and perceived importance of social media for social connection (*t_4151_*=6.85; *P*<.001). In terms of gender differences, males reported more daytime sleepiness (t_4111_=4.10; *P*<.001) and social media behaviors (checking: *t_4111_*=4.60, *P*<.001 and posting: *t_4111_*=4.15, *P*<.001) than females, whereas females reported greater interest and importance of using social media for these activities than males (*t_4111_*=3.58; *P*<.001). There were no differences in parents’ rules around bedtime screen or media use; however, those with parental rules tended to be younger. Table 1 presents the demographic characteristics and means for the primary study variables across the sample.

### Main Results

Our first study aim examined the direct effects of the frequency of social media checking and posting on daytime sleepiness. Consistent with the hypotheses, hierarchical linear regressions indicated that adolescents who checked and posted on social media more frequently had higher levels of daytime sleepiness (Table 2), covarying for age, gender, and age at first smartphone use. Social media posting and checking accounted for 7% and 8% more variance in daytime sleepiness, respectively.

For our analyses examining perceived social media importance and daytime sleepiness, we found that adolescents with greater perceived importance of social media for social interactions also reported higher levels of daytime sleepiness (Table 3), which accounted for 9% of the variance in daytime sleepiness. Among the covariates, younger adolescents and boys were also more likely to have higher levels of daytime sleepiness. Age at first smartphone use was not associated with daytime sleepiness.

**Table 2:** Social media posting and checking frequency and daytime sleepiness (N=4153).

Predictors	Pediatric Daytime Sleepiness Scale	
	Estimate, B (95% CI)	*P* value^a^
Intercept	12.49 (12.21 to 12.77)	<.001
Gender (1)^b^	1.07 (0.68 to 1.46)	<.001
Age	−0.26 (−0.39 to −0.13)	<.001
Phone age^c^	−0.01 (−0.10 to 0.07)	.76
Social media posting^d^	0.70 (0.63 to 0.78)	<.001
Social media checking^d^	0.76 (0.68 to 0.84)	<.001

^a^Statistically significant (*P*<.05).

^b^Gender: 0 (female) and 1 (male).

^c^Phone age: parent-reported age of adolescent first smartphone.

^d^Social media posting and checking reflect frequency on a daily basis. Models were conducted separately, with covariate estimates similar across models.

**Figure 1:**
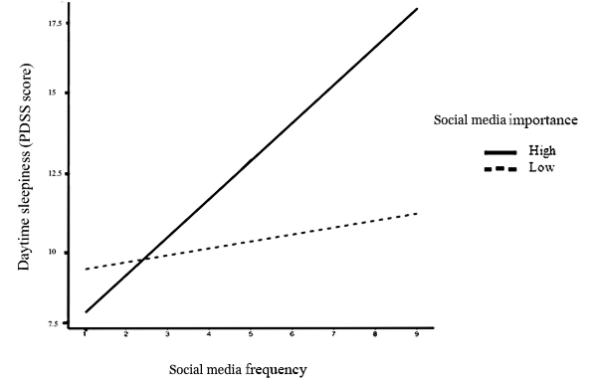
Effect of social media frequency on daytime sleepiness by levels of perceived social media importance. Daytime sleepiness scores were derived from the 8-item PDSS (Pediatric Daytime Sleepiness Scale).

For the moderation analyses, there was a significant interaction between social media frequency (average of checking and posting) and importance of social media for social interactions on daytime sleepiness (B=0.04, SE 0.007; *P*<.001), such that adolescents with more frequent social media use had higher levels of daytime sleepiness when they also had higher levels of social media importance for social interactions (B=0.76, SE 0.07; *P*<.001) than those with lower levels (B=0.29, SE 0.06; *P*<.001; Figure 1). Higher and lower levels reflect values one SD above and below the mean, respectively. The model with the interaction accounted for nearly 13% of the variance in daytime sleepiness, which is an additional 4% to 6% from prior models.

**Table 3:** Social media importance and daytime sleepiness (N=4153).

Predictors	Pediatric Daytime Sleepiness Scale	
	Estimate, B (95% CI)	*P* value^a^
Intercept	12.49 (12.21 to 12.77)	<.001
Gender (1)^b^	1.06 (0.67 to 1.44)	<.001
Age	−0.26 (−0.38 to −0.13)	<.001
Phone age^c^	−0.01 (−0.09 to 0.08)	.92
Social media importance^d^	0.36 (0.33 to 0.40)	<.001

^a^Statistically significant (*P*<.05).

^b^Gender: 0 (female) and 1 (male).

^c^Parent-reported age of adolescent’s first smartphone.

^d^Social media importance is the subscale (technology for social interaction) on Adolescents’ Digital Technology Interactions and Importance Scale.

**Figure 2:**
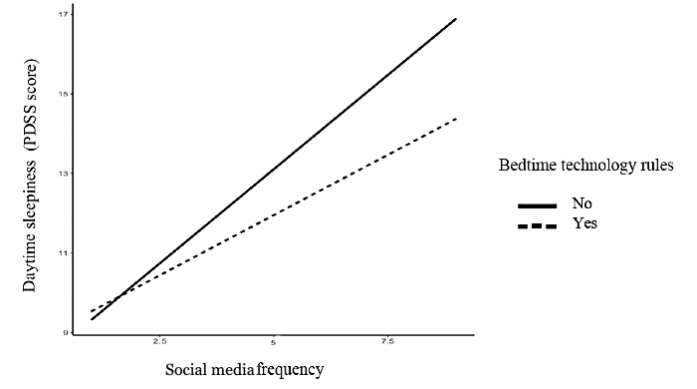
Social media frequency and daytime sleepiness by bedtime technology rules. Daytime sleepiness scores were derived from the 8-item PDSS (Pediatric Daytime Sleepiness Scale).

**Figure 3:**
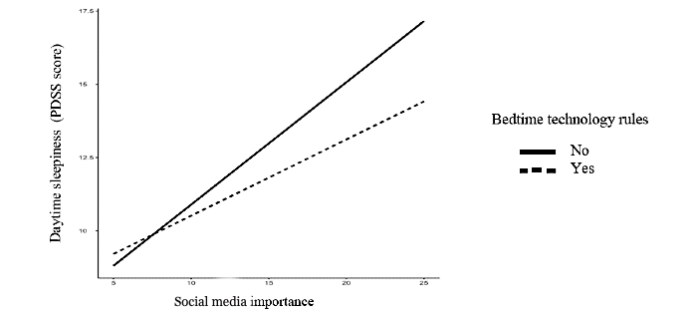
Social media importance and daytime sleepiness by bedtime technology rules. Daytime sleepiness scores were derived from the 8-item PDSS (Pediatric Daytime Sleepiness Scale).

The second moderation analysis indicated that there was a significant interaction between social media frequency and endorsement of parents’ technology rules around bedtime (B=−0.34, SE 0.09; *P*<.001). The relationship between social media frequency and daytime sleepiness was stronger for adolescents who did not have parental rules around bedtime (B=0.60, SE 0.07; *P*<.001) than for those who did have rules around bedtime (B=0.95, SE 0.05; *P*<.001; Figure 2). Similarly, there was a significant interaction between social media importance and bedtime technology rules (B=−0.16, SE 0.04; *P*<.001; Figure 3) on daytime sleepiness in the same direction.

For the moderation analyses examining specificity to bedtime technology rules, the results indicated that the interactions were not significant (with Bonferroni corrections) for social media frequency or social media importance and number of media access rules predicting daytime sleepiness.

## Discussion

### Principal Findings

Daytime sleepiness has significant consequences for adolescents’ mental health [6], including depression and suicide [9]. This study examined several potentially modifiable risk factors for daytime sleepiness: youth social media behaviors and perceptions and parental bedtime media rules. The results indicate that adolescents with more frequent social media checking and posting behaviors and those who value social media for social connection had higher levels of daytime sleepiness and those with both higher levels of social media use and importance were most likely to report daytime sleepiness. Furthermore, the absence of bedtime *screentime* rules exacerbated the effects of both social media frequency and importance on daytime sleepiness. Overall, our findings identify several modifiable behaviors among youth and their parents that affect youth sleepiness, with potential implications for adolescent mental health outcomes.

It is important to first describe behaviors around social media and technology in our sample, which provides a context for our findings and future research. In a large sample of adolescents aged 12-17 years representing the demographics of adolescents in the United States, 96% (3987/4153) of adolescents had their own smartphone, which was generally obtained by the of age 12. In general, nearly 1 in 5 adolescents reported checking their phones constantly, whereas more than 1 in 10 teens reported constantly posting on social media. Adolescents with more frequent social media behaviors also tended to have a greater perceived importance of social media for social belonging. Only 34.12% (1417/4153) of parents reported having rules in their household around bedtime screen use, and a total of 41.15% (1709/4153) of parents did not endorse having any rules around screen access (eg, certain rooms or times). Importantly, youth with no parental *screentime* rules around bedtime had more frequent social media behaviors and greater importance of social media for social connection than youth with parental rules.

Our findings suggest that more frequent social media behaviors and greater importance of social media are associated with higher levels of daytime sleepiness. Importantly, youth with both more social media use and who place more emphasis on social media for social purposes have higher levels of daytime sleepiness. Thus, even among those with more frequent social media use, social media importance further exacerbates this relationship, highlighting the importance of assessing youth perceptions of social media. It is possible that these adolescents (ie, those with more frequent social media use and perceived social media importance for social affordances) are more psychosocially affected by their experiences on social media, which may lead them to use even more social media and at times that may further delay or disrupt sleep [30]. Specifically, these adolescents may fear missing out on social experiences [29] when not using social media, and social media may displace other activities that promote wakefulness, such as exercise or in-person socializing. Furthermore, these youth may use social media more at night [31] to stay connected to peers, which increases exposure to blue light and psychophysiological arousal closer to bedtime [42]. Given that adolescents with higher levels of perceived social media importance also engage in more social media checking and posting activities, these youth may be in a vicious cycle. As youth use more social media and are more affected by it, they may have more sleep problems and daytime sleepiness, which, in turn, impairs youth mood and social interactions [43,44]. Less rewarding in-person peer interactions may then heighten youth’s perceptions that social media is important for social belonging, thereby contributing to a vicious cycle of acute and chronic sleepiness. Given that directionality cannot be determined from this cross-sectional study, further research is needed to examine these potential mechanisms and explore whether targeting youth social media behaviors and daytime sleepiness improves youth mental health.

Importantly, our study also examined whether parents’ media rules at bedtime affected the relationship between daytime sleepiness and social media frequency and importance. Consistent with prior research on parental bedtime monitoring [36] and rules around bedtime technology use [39], the absence of *screentime* rules around bedtime exacerbated the effects of both social media frequency and importance on daytime sleepiness. Specifically, adolescents with more social media use and perceived importance had higher levels of daytime sleepiness if their parents did not have rules for bedtime technology use. These findings suggest that parents’ rules at night may protect against the effects of social media on sleepiness. However, it is important to note that parents who have technology rules at bedtime may have additional bedtime rules or more parental monitoring of social media behaviors that affect sleepiness. There might also be a *third variable* of parenting or parent-child relationships influencing these associations, such as how parents themselves use and relate to social media, which may affect both enforcement of rules and teens’ social media use [45]. Of note, we did not find a moderating effect of total *screentime*, suggesting specificity of bedtime media rules on social media and sleep outcomes. Future research should not only examine a greater scope of parental mediation to better understand which parenting behaviors have the greatest buffering effect on social media and specific youth outcomes but also explore other factors that moderate the association between parental rules and social media use and ultimately daytime sleepiness. For instance, it is possible that media rules at bedtime not only affect youth sleep but also protect youth from using social media in a way that exacerbates their mood and anxiety, which should be examined in future studies. However, individual differences among adolescents may affect their need for specific technology use rules; youth with poorer self-regulation may benefit more from parental rules around bedtime technology use than others [46].

### Limitations

Although our study provides preliminary data suggesting the importance of examining individual and environmental factors involving social media, it is not without limitations. First, our study is cross-sectional, which limits our ability to determine causality. It is possible that adolescents with sleepiness and poor mental health may be more likely to use social media [47], and this is likely a bidirectional relationship that fuels a vicious cycle for certain youth. Second, this study did not examine sleep and circadian characteristics that likely mediate and further explain the observed associations between social media and daytime sleepiness. Future research is needed to examine these relationships using both objective and subjective methods as well as multi-informant approaches to daytime sleepiness and other sleep characteristics. Our study did not evaluate other aspects of social media, such as actual stressors or positive and negative peer interactions or adolescents’ quality of peer relationships and perceived importance of social feedback more generally, which may provide more information about the specificity of the observed associations. Our study also did not define social media for youth, which might affect how teens responded to social media checking and posting questions. However, given individual differences and rapid changes in social media, capturing youth-defined posting and checking behaviors on social media may better capture youth’s individual experiences. Furthermore, we did not examine youth pubertal development, which may affect youth perception and engagement in social media [48] and daytime sleepiness [5]. Thus, it is important to consider puberty and explore potential gender differences in these associations. Although our study examined whether parents had bedtime rules around technology use, we did not test the enforcement or effectiveness of these rules, which is an important extension of this study. Finally, our study did not assess whether sleepiness was associated with poor mental health outcomes, which is critical in further highlighting the importance of this study in mental health prevention efforts.

### Conclusions

The findings from this study provide initial support for the associations between daytime sleepiness and both social media use and perceived importance of social interactions in a large, nationally representative sample of adolescents. Although specific to daytime sleepiness, our results have important implications for adolescent mental health more broadly. First, daytime sleepiness is significantly associated with and prospectively predicts youth mental health problems [1], including depression and suicide [9], which are major public health crises. Second, this study highlights the importance and potential clinical relevance of targeting social media use and advances our understanding of which youth may be most at risk for negative effects of social media (eg, those who highly value social media use for social belonging), which may also extend to other mental health outcomes. Finally, our study indicates the importance of parental rules around media use on youth behaviors and outcomes, which suggests that parenting behaviors may affect how and in what ways youth use social media and its potential effects on their mental health. Thus, parents and youth providers (eg, clinicians, school counselors, and educators) should consider discussing parental rules around technology use with families, particularly around bedtime. Given that parenting rules are modifiable, these results may have important implications for interventions aimed at improving social media use and protecting adolescents from its negative effects on mental health. Although preliminary, these findings may be beneficial for researchers and clinicians in the development and implementation of prevention and intervention programs for social media, sleep, and mental health.
